# Co‐creating and implementing a novel pre‐conference event to promote equity and inclusivity among academic researchers and people who use drugs

**DOI:** 10.1111/dar.13882

**Published:** 2024-05-27

**Authors:** M. J. Stowe, Louise Hansford, Rachel Halford, Jason Wallace, Lise Lafferty

**Affiliations:** ^1^ The Kirby Institute UNSW Sydney Sydney Australia; ^2^ The South African Network of People Who Use Drugs (SANPUD) Cape Town South Africa; ^3^ Midlands Partnership Foundation Trust, National Health Service Stafford UK; ^4^ The Hepatitis C Trust London UK; ^5^ Scottish Drugs Forum Glasgow UK; ^6^ Centre for Social Research in Health UNSW Sydney Sydney Australia

**Keywords:** conferences, people who use drugs, early‐ and mid‐career researchers, inclusivity, networking

## Abstract

This commentary draws on our experience organising a targeted networking event at the 10th International Conference on Health and Hepatitis in Substance Users, in Glasgow, Scotland in October 2022. The event, held the day before the conference, brought together people with lived and living experiences of drug use and early‐ and mid‐career researchers on an equitable basis. We offer reflections, focussing on how the event promoted community‐academic engagement from members of the respective groups. We provide recommendations for how conferences can organise to engage with people who use drugs—both those with lived and living experience and foster greater inclusion for all attendees.

## 1 EQUITY AND INCLUSIVITY AT ACADEMIC CONFERENCES

Conferences centred around the health and human rights of people who use drugs across key populations are ideal environments to foster and establish community‐academic relationships [1]. However, structural barriers and power differentials work to exclude those who have historically been marginalised [2, 3]. As a result, people who use drugs are often not empowered to partner with researchers and are excluded from research priority setting discussions and, consequently, the co‐production of knowledge [4].

Since the early 1990s, drug user activists have mobilised to attend national and international health and drug‐use‐related conferences [5]. This has resulted in the establishment of drug user networks and funding for programmes that provide harm reduction and health care services [6, 7], though meaningful inclusion at conferences is yet to be fully recognised. While relentless and persistent effort from activists and allies have helped shift the balance between power and participation at conferences, continued effort is needed to ensure that people who use drugs are not only present in conference spaces but are also able to meaningfully participate and engage [8, 9].

People who use drugs often represent ‘the community voice’, invited to present and publicly share their personal stories [8], rather than be engaged and recognised throughout the conference as living experts with valuable insights for research translation, and also as knowledge holders during research priority setting. This exacerbates the knowledge divide and recognition of people with lived and living experience of drug use as equals in the production of knowledge. Even when people who use drugs are included in conferences, there is a critical power imbalance, whereby expertise is recognised in qualifications, rather than first‐person lived experience. In this way, academics continue to perpetuate their position as the holders of the power [10], further entrenching divides, forcing an ‘us and them’ scenario that is serving no one.

For early and mid‐career researchers, conferences are often expected to be an opportunity to build their research profile, to ‘get their name out there’ and build connections. Early‐ and mid‐career researchers typically arrive at the conference with their organisational cohort, glued together, relying on supervisors and other academics from within their university to introduce them to others, all in the name of networking. But a critical gap is missed for early‐ and mid‐career researchers to connect with experts in—and among—the community and to foster in a new era of research in which co‐design can occur where it is intended—from the inception stage, rather than down the track when the study has already been conceived and the protocol written.

With all of this in mind, we set out to facilitate a networking event between people who use drugs and early‐ and mid‐career researchers at the 10th International Conference on Health and Hepatitis in Substance Users in Glasgow, Scotland.

## 2 THE CONFERENCE

The International Network on Health and Hepatitis in Substance Users (INHSU) is a global organisation dedicated to scientific knowledge exchange, knowledge translation and advocacy focused on drug‐related social and health harms [11]. INHSU held its first scientific meeting in 2009, with conferences held biannually until 2015 when the conference progressed to annual meetings.

Bringing together experts from around the globe, the annual INHSU conferences facilitate dissemination of the latest advances in viral hepatitis, including epidemiology, management and treatment of viral hepatitis among people who use drugs, and look at new aims which move beyond hepatitis C and includes the overall health and harms that may be associated with drug use. Initially focused on viral hepatitis, INHSU has broadened its scope to include other infectious diseases and drug‐related harms experienced by people who use drugs [11].

In 2022, INHSU held the 10th International Conference on Health and Hepatitis in Substance Users in Glasgow, Scotland from 19 to 21 October, and included 811 registered participants comprising healthcare practitioners, researchers, policymakers, advocates, representatives from government and non‐government organisations, and community members, including people who use drugs.

## 3 CONFERENCE COMMITTEES

As part of the organising for the annual conference, INHSU established three sub‐committees to support development of content relevant to the groups represented. These committees are Nurses (often frontline providers of hepatitis C care), Community (inclusive of people with lived and living experience of drug use) and Early‐ and Mid‐Career Researchers (conducting research into health of people who use drugs). Members of the latter two collaborated to develop and implement a targeted in‐person event the day prior to the start of the conference.

## 4 THE PRE‐CONFERENCE EVENT

Multiple engagements between Community and Early‐ and Mid‐Career Researcher Committee representatives resulted in the decision to develop a targeted, community‐initiated networking event as a mechanism to foster connections between Community Members and Early‐ and Mid‐Career Researcher members prior to and throughout the conference. The event was designed to unite the growing interest of health professionals, academics, and communities in giving communities a genuine voice in research, and therefore to increase the likelihood of successful research translation.

A working group was developed, comprising the five authors of this commentary with three members from the United Kingdom, one from Australia and one from South Africa with diverse experiences of research and personal histories of drug use. The working group then met semi‐regularly over the following 5 months to develop and shape the idea of the networking event, including securing external funding.

Approximately 65 people attended the connecting event, with roughly 35 people from the community and 30 early‐ and mid‐career researchers. We believe the high attendance numbers indicate strong interest in opportunities among living and lived experience and academic attendees to connect, particularly in sessions specifically designed for networking and collaboration.

The networking event was designed to pair community members with academics. During the event, Community Members were invited to stand in a line opposite a line of Early‐ and Mid‐Career Researchers allowing for simple pairing to occur across the two groups, ensuring each pairing included a Researcher and a Community Member. Pairs were then given 2 min to ask each other questions, then rotate to the next person down the line. A list of questions was developed among the working group members and provided to attendees as a ‘back up’ should pairs get stuck for conversation topics. This orchestrated portion of the session was anticipated to last for approximately 30–40 min, allowing for multiple connections to occur, before progressing into unstructured networking. Though the shape of room hindered the flow of movement and rotation pairing, the space was abuzz with conversation with people moving throughout and actively engaging with one another making new connections.

The event was scheduled at the end of the Community Day—a pre‐conference event planned by and for members of the drug user community—and so participants from the Community Day were invited to stay and get involved, many of whom did. At the start of the event, we outlined the format and went through confidentiality and boundaries around personal questions (i.e., being sensitive and respectful about the questions being asked as, e.g., questions about family may be difficult for anyone for a variety of reasons). However, we also acknowledged all participants (i.e., community and early‐and‐mid career researchers) were adults and could take responsibility for what they did and did not share.

To our knowledge, at least one pairing occurring during the event has led to ongoing research collaborations in the time since.

## 5 LESSONS LEARNED

The connection event taught us valuable lessons and provided us with unique insights into how engagements between Early‐ and Mid‐Career Researchers and Community Committees can take place and inform future conference events going forward. Event attendees were invited to ‘write down a few words’ about how they felt/viewed/perceived the event on a small blank piece of paper prior to their departure. Participant reflections indicated appreciation of the event and opportunity to connect with others in an inclusive way, with several attendees requesting the event be replicated at future conferences.

The event taught the authors valuable lessons and provided unique insights into how engagements between Community and Early‐ and Mid‐Career Researcher Committees can take place and inform future conference events going forward. Due to earlier attendance at the Community Day, Community Members arrived with name tags, creating a visual ‘outing’ of people who use drugs – an issue easily remedied with name tags for everyone.

Through planning and implementation of this novel networking event, we had several key learnings to take forward to future networking events:

1. A targeted networking event should be integrated early into the conference, if not immediately prior, so attendees have greater opportunity to connect throughout the conference. Use of encrypted messenger applications to create groups, facilitated conversation through the conference and ongoing connections.
2. Confidentiality and boundaries need to be established, with all attendees having the right to engage/participate as much as each individual feels comfortable.
3. Conversations were organic, with few prompts or suggested questions required. Five‐minute pairings (rather than the originally planned 2‐min connections) enabled time for discussion and connecting while maintaining the fast‐paced atmosphere.
4. Greater engagement with potential attendees through the respective committees may facilitate a more responsive event and foster greater participation.
5. A flexible and responsive approach helps ensure that unforeseen challenges and complexities are managed effectively.

However, it would be remiss of us to overlook the reality that the networking event was not accessible to everyone. The noise levels were loud owing to room acoustics which may have been a barrier for those with sensory needs. Given the high movement within the room to swap conversation partners, this may not have been suitable for people with difficulties standing over long periods. While chairs were available along the periphery of the room, this may have unintentionally excluded or marginalised those requiring seating during the session. Additionally, there may have been further barriers for broader conference attendance, such as visas and financial limitations, which are beyond the scope of the networking event. Some attendees at the earlier Community Day event indicated a preference not to stay given this would be a long day (having just spent several hours in a session), though many people did return for the networking event. It may be that the short break between events facilitated enough time for people to step away and return. In light of these remaining barriers, the event surpassed all of our expectations with the groups connecting meaningfully and being very responsive to the spirit of the event.

## 6 RECOMMENDATIONS

Innovative meeting formats that maximise participation and promote partnerships help make conferences less inhibiting and create spaces for people who use drugs who have historically been marginalised. Inclusive environments better ensures that delegates with lived/living experience are empowered to shape the debate and set the agenda. This can be achieved through:

1. Involving people with living and lived experience in development of conference programmes and identification of invited/keynote speakers;
2. Reducing attendance barriers for people with living and lived experience through availability of facilities for self‐care, scholarships and other initiatives, such as opportunities to connect with others;
3. Engaging with people with living and lived experience in clinically focused conferences can help delegates to understand problems that matter most to clients/patients (and, consequently, their health care providers); and
4. Engaging with people with living and lived experience can foster collaborations in health care design, education, research and clinical care improvements—all of which are critical to improving the health and wellbeing of people who use drugs.
5. Conference role‐players being open to different more inclusive meeting formats, such as unconferences. Unconferences are non‐hierarchical, participant driven, self‐managed meeting formats which seek to avoid top‐down hierarchical knowledge transmission found within traditional conferences [12]. Events (such as unconferences) which are equity‐focused and designed to be inclusive, rather than elitist, require trust and empowerment of co‐organisers to tailor and develop meaningful events [13]. At INHSU, this has been, for the most part, achieved through facilitation of the Community Day the day immediately prior to the start of the conference.
6. Conference attendees, particularly at meetings in which community representation occurs and in which power imbalances may be further exacerbated, be mindful of any implicit biases they may bring and how their interactions—through networking, presentations, Q&As—may affect the sense of safety of all delegates.
7. Lastly, and perhaps most importantly, conference organisers have a responsibility to facilitate connection for all attendees in a safe and collaborative space. As such, conference organisers should consider environmental spaces and inclusivity, considering that elite venues such as 5‐star hotels, can create an environmental barrier for some delegates.

While our experience—and by extension this commentary—focuses on representation and inclusion of people with living and lived experience of drug use, the inclusive strategies outlined can be utilised in any conference in which people with lived experience of any type contribute to, participate in, and attend.

## 7 CONCLUSIONS

We reflect on our experiences as conference attendees with living and lived experience and allies in academia to highlight the potential for targeted and socially responsive conference activities to foster community‐academic partnerships and reduce power imbalances throughout the research continuum. As people who use drugs with lived‐ and living experience, and allies, we believe inclusivity is an ambition that organisers can and should continuously work towards, always allowing room for learning and improvement. Failure to implement these recommendations will continue to stigmatise and marginalise attendees from impacted communities.

## AUTHOR CONTRIBUTIONS

All authors significantly contributed to the planning and implementation of the networking event reported within this commentary. All authors contributed to the conceptualisation, drafting and review of this manuscript.

## CONFLICT OF INTEREST STATEMENT

Lise Lafferty has received speaker fees from AbbVie unrelated to this work.

## References

[1] Jozaghi E , Greer AM , Lampkin H , Buxton JA . Activism and scientific research: 20 years of community action by the Vancouver area network of drug users. Subst Abuse Treat Prev Policy. 2018;13:18. 29788975 10.1186/s13011-018-0158-1 PMC5964704

[2] Damon W , Callon C , Wiebe L , Small W , Kerr T , McNeil R . Community‐based participatory research in a heavily researched inner city neighbourhood: perspectives of people who use drugs on their experiences as peer researchers. Soc Sci Med. 2017;176:85–92. 28135693 10.1016/j.socscimed.2017.01.027 PMC5438752

[3] Simon C , Brothers S , Strichartz K , Coulter A , Voyles N , Herdlein A , et al. We are the researched, the researchers, and the discounted: the experiences of drug user activists as researchers. Int J Drug Policy. 2021;98:103364. 34294521 10.1016/j.drugpo.2021.103364 PMC9759691

[4] Souleymanov R , Kuzmanović D , Marshall Z , Scheim AI , Mikiki M , Worthington C , et al. The ethics of community‐based research with people who use drugs: results of a scoping review. BMC Med Ethics. 2016;17:25. 27129927 10.1186/s12910-016-0108-2 PMC4850694

[5] Jürgens R . “Nothing about us without us”—greater, meaningful involvement of people who use illegal drugs: a public health, ethical, and human rights imperative, International edition. Toronto: Canadian HIV/AIDS Legal Network, International HIV/AIDS Alliance, Open Society Institute: Canadian HIV/AIDS Legal Network, International HIV/AIDS Alliance, and Open Society Institute. 2008.

[6] Efthimiou‐Mordaunt A . Junkies in the House of the Lord. Subst Use Misuse. 2015;50:1159–1164. 26361921 10.3109/10826084.2015.1017336

[7] Brooks HL , Husband C , Taylor M , Sherren A , Hyshka E . Supporting the full participation of people who use drugs in policy fora: provision of a temporary, conference‐based overdose prevention site. Int J Drug Policy. 2020;84:102878. 32739614 10.1016/j.drugpo.2020.102878

[8] Byrne J , Albert ER . Coexisting or conjoined: the growth of the international drug users' movement through participation with international harm reduction association conferences. Int J Drug Policy. 2010;21:110–111. 19942422 10.1016/j.drugpo.2009.10.009

[9] Temenos C . Mobilizing drug policy activism: conferences, convergence spaces and ephemeral fixtures in social movement mobilization. Space Polity. 2016;20:124–141.

[10] Fook J . Social work: a critical approach to practice. 3rd ed. London: Sage Publications Ltd; 2016.

[11] International Network on Health and Hepatitis in Substance Users . Working together to improve the health of people who use drugs. Sydney: INHSU; 2023.

[12] King D , Griffin M , Bell E . Inclusion and exclusion in management education and learning: a deliberative approach to conferences. Acad Manag Learn Edu. 2023;22:40–62.

[13] Budd A , Dinkel H , Corpas M , Fuller JC , Rubinat L , Devos DP , et al. Ten simple rules for organizing an unconference. PLoS Comput Biol. 2015;11:e1003905. 25633715 10.1371/journal.pcbi.1003905 PMC4310607

